# The Effect of Bifidobacterium on Reducing Symptomatic Abdominal Pain in Patients with Irritable Bowel Syndrome: A Systematic Review

**DOI:** 10.1007/s12602-019-09609-7

**Published:** 2019-11-18

**Authors:** Charlotte Pratt, Matthew D. Campbell

**Affiliations:** School of Food Science and Nutrition, University of Leeds, Leeds, UK

**Keywords:** Bifidobacterium, Irritable bowel syndrome, Abdominal pain

## Abstract

Probiotics, specifically *Bifidobacteria*, may improve abdominal pain in patients with irritable bowel syndrome (IBS); however, results from randomised controlled trials (RCTs) are conflicting. Here, we systematically reviewed the efficacy of *Bifidobacteria* on abdominal pain in IBS. We searched MEDLINE, EMBASE and the Cochrane Controlled Trials Register from inception to 20 May 2019, without language or date restrictions. The search strategy comprised of the combination of three concepts: supplementation, abdominal pain, and IBS. Inclusion criteria included double-blind placebo-controlled RCTs featuring *Bifidobacteria* supplementation in Rome-diagnosed IBS patients. A total of 8 RCTs involving a total of 1045 patients with Rome diagnosed IBS were included. The dose of total *Bifidobacteria* ranged from 10^6^ to > 10^11^ cfu (colony-forming unit) and duration of supplementation ranged between 2 and 8 weeks. *Bifidobacteria* was delivered through either intake of fermented milk products, encapsulation or via a malted milk beverage, with all studies assessing abdominal pain via a visual analogue Likert scale. From the studies included, 50% (n = 4) of studies found a statistically significant improvement in abdominal pain following *Bifidobacteria* supplementation compared to placebo, 38% (n = 3) of studies found non-significant improvements and 12% (n = 1) showed a statistically significant dose-response effect of improvement. The evidence shows a heterogeneity of effect for *Bifidobacteria* dependent upon strain, dosage and delivery method. While not all studies demonstrate significant improvements in abdominal pain, none of the selected studies reported an increase in pain or other adverse effects.

## Introduction

Irritable bowel syndrome (IBS) is one of the most widely recognised functional bowel disorders globally, characterised by chronic or recurrent abdominal pain [1]. While it is a common condition, the aetiology is not fully understood. However, classical hallmarks include disturbances in gut microbiota, low-grade mucosal inflammation, immune activation and altered intestinal permeability [2]. Probiotics, which are live microbial supplements that colonise in the colon and serve to modulate the intestinal microbial-inflammatory-immunological milieu, have been shown to yield beneficial effects on both the clinical course and symptoms of IBS [3, 4]. Probiotics are numerous and exert divergent effects depending upon the unique characteristics of their composition, namely, their genus, species and strain [5]; while some probiotics display a desired anti-inflammatory effect, others principally impact motility [6] and visceral sensation [7]. Thus, the efficacy of probiotic supplementation on reducing symptoms of IBS is largely dependent upon, and specific to, the individual probiotic used.

Much of the research has investigated the impact of composite probiotic mixtures containing several strains of *Lactobacillus, Bifidobacterium* and *Propionibacterium*, which hampers the assessment of specific strains in relieving IBS symptoms. In IBS, patients typically present with significantly lower levels of *Bifidobacteria* in faecal and duodenal mucosa samples, yet other major bacterial groups remain preserved [4]. Evidence suggests that *Bifidobacteria* supplementation, which serves to restore a balanced microbial composition, modulates immune function, gut microbiota and intestinal mucosal adhesion in IBS patients [8], with studies demonstrating positive effects on epithelial cell adherence, reinforcement of tight junctions, stimulation of IgA production and cell-mediated immunity, which are impaired in IBS patients [9, 10]. Based on these promising findings, clinical trials have been conducted to establish whether *Bifidobacteria* supplementation reduces abdominal pain in IBS; however, results are conflicting. Therefore, this investigation aimed to systematically review human studies in which the efficacy of *Bifidobacteria* supplementation had been examined as a treatment for abdominal pain in IBS.

## Methods

This systematic review was conducted in accordance with PRISMA (Preferred Reporting Items for Systematic Reviews and Meta-analyses) guidelines [11] and prospectively registered. A search of the medical literature was conducted using MEDLINE, EMBASE and the Cochrane Controlled Trials Register from inception to 20 May 2019, without language or date restrictions, for all RCTs investigating the impact of *Bifidobacteria* on abdominal pain in patients with IBS. The search strategy comprised of the combination of three concepts: supplementation, abdominal pain and IBS. For these three items, relevant keyword variations were used, which included keyword variations in the controlled vocabularies of the different databases, as well as free text word variations. Inclusion criteria included double-blind placebo-controlled RCTs featuring *Bifidobacteria* supplementation in Rome-diagnosed IBS patients.

Two investigators (CP and MDC) independently reviewed all RCTs by title and abstract and subsequently by full-text evaluation. Any disagreements were resolved through arbitrations with a third researcher independent from the research team. If relevant study information was missing, authors were contacted, and foreign language manuscripts translated where necessary. The bibliographies of all identified studies were used to perform a recursive search of the literature. The quality of the articles was assessed by the two independent reviewers according to the levels of evidence and the recommendations used for good clinical practice [12]. Risk of bias was assessed using the Cochrane Collaboration Risk of Bias tool. Each RCT was given one of three rankings, ‘high risk’, ‘low risk’, or ‘unknown risk’, in each of the following domains: sequence generation, allocation concealment, blinding of participants and personnel, blinding of outcome assessors, incomplete data, selective outcome reporting, and other sources of bias. Discrepancies which arose during this process were resolved firstly by discussion then by a third independent researcher where necessary. Risk of bias outcomes is presented within Supplement 1, Table 1.

**Table 1:** Trial characteristics and outcomes of RCTs investigating the impact of *Bifidobacteria* supplementation on reducing abdominal pain in IBS patients

Author	Subjects	Population	Probiotic	Dose	Duration	Measurement of abdominal pain	Outcome
Agrawal et al. 2008 [13]	34	Aged 20–69 years, female	*Bifidobacterium lactis* DN-173 010	1.25 × 10^10^	4 weeks	Daily symptom recorded on a 6-point Likert scale	Pain in treatment group reduced significantly (*P* = 0.044) compared to pain in control group
Biviano et al. 2017 [10]	34	Aged 18–65 years, mixed gender	*Bifidobacterium longum* BB-536 and Lactoferrin	3 × 10^9^	2 weeks	Symptom recorded on a 100-point Likert scale for last 10 days of treatment	Pain in treatment group reduced significantly (*P* < 0.007) compared to pain in control group
Charbonneau et al. 2013 [14]	61	Aged 18–65 years, mixed gender	*Bifidobacterium longum infantis* 35624	1 × 10^9^	8 weeks	Daily symptom recorded on a 6-point Likert scale through weeks 4 and 8 of the treatment period	No significant difference between pain in treatment and control group (*P* = 0.261), improvement from baseline similar in both groups
Guglielmetti et al. 2011 [15]	103	Aged 18–65 years, mixed gender	*Bifidobacterium bifidum* MIMBb75	1 × 10^9^	4 weeks	Daily symptom recorded on a 7-point Likert scale	Pain in treatment group reduced by a score of− 0.82 compared to baseline, significant reduction (*P* < 0.0001) in pain every week compared to the control group
Guyonnet et al. 2007 [16]	267	Aged 18–65 years, mixed gender	*Bifidobacterium animalis*	1.25 × 10^10^	6 weeks	Pain assessed at baseline and at the end of weeks 3 and 6 on a 6-point Likert scale	Pain at weeks 3 and 6 reduced from baseline in both groups (*P* < 0.001), but difference between groups not significant
Min et al. 2012 [17]	117	Aged 18–70 years, mixed gender	*Bifidobacterium lactis* and acacia fibre	> 10^11^	8 weeks	Pain assessed at baseline and at the end of week 8 on a 100-point Likert scale	Pain in IBS-D treatment group reduced (approaching significance; *P* = 0.05) compared to pain in control group but insignificantly in the all patient group (*P* = 0.26)
O’Mahony et al. 2005 [6]	67	Aged 18–75 years, mixed gender	*Bifidobacterium longum infantis* 35624	1 × 10^10^	8 weeks	Daily symptom recorded on a 7- point Likert scale as well as on a 10-point Likert scale	Pain in treatment group reduced significantly (*P* < 0.05) compared to pain in control for most weeks of the treatment
Whorwell et al. 2006 [18]	362	Aged 18–65 years, women	*Bifidobacterium longum infantis*	1 × 10^6^ 1 × 10^8^ 1 × 10^10^	4 weeks	Daily symptom recorded on a 6-point Likert scale	Pain reduced significantly (*P* = 0.023) in 1x10^8^ cfu treatment group compared to control group, other doses did not improve significantly (*P* = 0.24; *P* = 0.44)

## Results

**Fig. 1:**
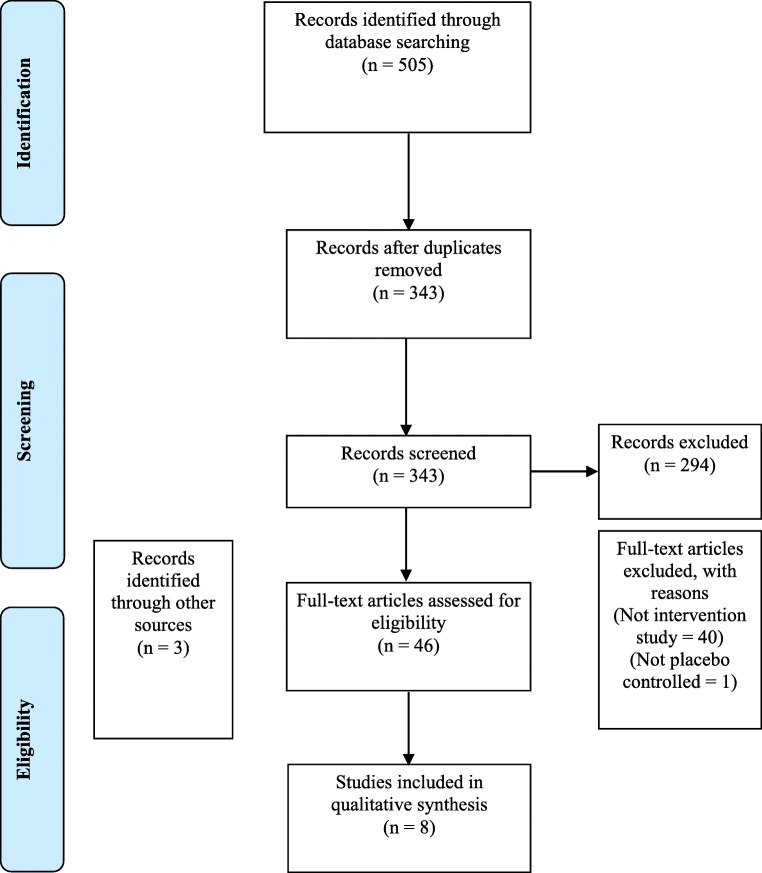
Flowchart of trial selection

In total, 343 records were identified through database screening, of these 8 RCTs were eligible and included in this review (Fig. 1; Table 1). A total of 1045 adults aged between 20 and 75 years with Rome-diagnosed IBS were included. The ranges of doses of total *Bifidobacteria* were 10^6^ to > 10^11^ cfu and duration of supplementation between 2 and 8 weeks. *Bifidobacteria* was delivered through either intake of fermented milk products (n = 3) [13, 16, 17], encapsulation (n = 4) [10, 14, 15, 18], or via a malted milk beverage (n = 1) [6], with all studies assessing abdominal pain via a visual analogue Likert scale.

From the studies included, 50% (n = 4) of studies found a statistically significant improvement in abdominal pain following *Bifidobacteria* supplementation compared to placebo, 38% (n = 3) of studies found non-significant improvements, and 12% (n = 1) showed a statistically significant dose-response effect of improvement (Table 1).

### Enriched Fermented Milk Products

Agrawal and colleagues [13] investigated trialled *Bifidobacterium lactis* in a fermented milk product consumed daily over a 4-week intervention compared to a daily probiotic-naive placebo non-fermented product. Individuals under the treatment condition were found to have significantly lower levels of abdominal pain compared to the control group. Guyonnet and colleagues [16] investigated the effect of *Bifidobacterium animalis* in a yoghurt compared to a placebo heat-treated yoghurt. Abdominal pain improved equally in both the treatment and placebo group overtime at 3 and 6 weeks compared to baseline; no conditional differences between treatment and placebo were evident. Similarly, Min and colleagues [17] investigated the use of daily intake of yoghurt enriched with *Bifidobacterium animalis subspecies lactis*. This placebo-controlled trial compared symptoms of abdominal pain following an 8-week treatment phase and failed to yield statistically significant benefits. Of note, the placebo in this trial consisted of a traditional yoghurt, which is likely to contain traces of *Bifidobacteria*, albeit in significantly lower amounts, which may account for the null effects on abdominal pain between the two conditions. Furthermore, the interventional treatment contained acacia gum, a complex indigestible polysaccharide fermented in the colon, which may confound potential improvements in abdominal pain resulting from *Bifidobacteria* alone.

### Encapsulation

Guglielmetti and colleagues [15] trialled daily encapsulated delivery of *Bifidobacterium bifidum* against a placebo control over a 4-week treatment period. The authors observed significant time-course reductions in subjective abdominal pain from weeks 1 to 4, which persisted into the subsequent 2-week washout period. Charbonneau et al. [14] investigated the effect of encapsulated delivery of daily *Bifidobacterium longum subspecies infantis* over an 8-week period and reported reduced levels of perceived abdominal pain at the end of the supplementation period. Similarly, these findings are supported by those of Biviano et al. [10] in which 2-week daily supplementation of *Bifidobacterium longum and* Lactoferrin was trialled, resulting in a significant reduction in pain scores in the treatment group compared to control. Of note, this study included Lactoferrin, a prebiotic for *Bifidobacterium* into the treatment product; further research is needed to establish whether the Lactoferrin in addition to *Bifidobacterium* in isolation carries any additive effect on abdominal pain or adjunct effects on other symptoms of IBS. Whorwell et al. [18] investigated the dose-response of encapsulated *Bifidobacterium infantis* supplementation whereby subjects were randomly allocated to receiving either a daily capsule of *Bifidobacterium infantis* at a dosage of 1 × 10^6^, 1 × 10^8^ or 1 × 10^10^ cfu or placebo control for 4 weeks. The authors reported a beneficial treatment effect for *Bifidobacterium infantis* at a dosage of 1 × 10^8^ cfu which persisted throughout the subsequent two-week washout period, while dosages of 1 × 10^6^ or 1 × 10^10^ cfu did not yield significant reductions in abdominal pain.

### Malted Milk Beverage

O’Mahony and colleagues [6] investigated the impact of *Bifidobacteria infantis* on abdominal pain delivered through a daily malted milk beverage over a course of 8 weeks. Significant time course improvements were observed following treatment, with improvements in abdominal pain evident at week-1, peaking at week-2, with a sustained suppression of symptoms up to trial cessation at 8 weeks.

## Discussion

There is conflicting evidence regarding the clinical utility of probiotics, specifically *Bifidobacteria*, in reducing subjective abdominal pain in patients with IBS. Our systematic review provides the most comprehensive and contemporary review to date assessing the impact of *Bifidobacteria* on abdominal pain in patients with IBS. Considering the cumulative findings from 8 RCTs with a total of 1045 adults with IBS, we conclude that evidence shows a heterogeneity of effect for *Bifidobacteri*a dependent upon strain, dosage and delivery method. Importantly, while not all studies demonstrate a statistically significant improvement in abdominal pain, none of the selected studies in this review reported an increase in pain or other adverse effects compared to placebo.

Irritable bowel syndrome (IBS) is one of the most widely recognised functional bowel disorders globally, characterised by chronic or recurrent abdominal pain [1]. In the UK, ~ 17% of the general population live with IBS, and up to 50% of all general practitioner (GP) visits for gastrointestinal disturbances relate to IBS symptoms [19]. IBS poses a significant personal, societal and economic burden [20–22], thus clinically effective and pragmatic treatment options which serve to reduce the symptoms of IBS are much needed. Although several reviews have previously concluded that probiotics improve IBS symptoms including abdominal pain [23–28], many reviews feature studies investigating the use of composite probiotic mixtures containing several strains of *Lactobacillus, Bifidobacterium* and *Propionibacterium*, which hampers the assessment of specific strains in relieving IBS symptoms. As such, this is the first study to systematically review the clinical effectiveness of single strains of *Bifidobacterium* on symptomatic abdominal pain in patients with IBS. Despite applying stringent inclusion criteria and rigorous methodology, there are several concerns regarding the quality of available evidence. Firstly, the available literature lacks uniformity in the *Bifidobacterium* species tested and the dosage used. From the investigations reviewed in this study, three differing strains of *Bifidobacteria* were sampled, and dosages varied from 10^6^ to > 10^11^ cfu. Secondly, the delivery of the probiotics varied between studies, including delivery via fermented milk products [13, 16, 17], a malted milk beverage [6] or via encapsulation [10, 14, 15, 18]. Thirdly, treatment periods varied from 2 weeks to 8 weeks with variable washout periods; considering the intermittent nature of symptomatic IBS abdominal pain, short treatment durations and short observation windows may fail to adequately capture the effect, and legacy effect, of supplementation or severity of symptoms. Fourthly, the composition of placebos used in some studies may confound study findings [10, 17]. In future studies, it would be helpful to stratify IBS patients by clinical presentation and/or by severity of symptoms, and include longer treatment durations and longer wash-out periods to establish long-term legacy effects and whether dosages can be taken less frequently.

Currently, the evidence shows a heterogeneity of effect for *Bifidobacteria* on abdominal pain in IBS dependent upon strain, dosage and delivery method. Future research should consider investigating whether divergent treatment responses are determined by supplementation characteristics (strain, dosage and delivery method), as well as individual clinical parameters which may influence treatment effectiveness (e.g. gut microbiota composition, gut motility), and whether these clinical parameters are useful tools for predicting treatment outcomes. This review should help others obtaining a balanced view of the relevant literature available. Presently, it is difficult to strongly advocate the use of *Bifidobacteria* for reducing abdominal pain in IBS; however, as none of the selected studies in this review reported an increase in pain or other adverse effects, we can at least have a greater degree of confidence in remarking that *Bifidobacteria* supplementation is unlikely to have an adverse effect.
